# Vitamin D Metabolic Pathway Genes and Pancreatic Cancer Risk

**DOI:** 10.1371/journal.pone.0117574

**Published:** 2015-03-23

**Authors:** Hannah Arem, Kai Yu, Xiaoqin Xiong, Kristin Moy, Neal D. Freedman, Susan T. Mayne, Demetrius Albanes, Alan A. Arslan, Melissa Austin, William R. Bamlet, Laura Beane-Freeman, Paige Bracci, Federico Canzian, Michelle Cotterchio, Eric J. Duell, Steve Gallinger, Graham G. Giles, Michael Goggins, Phyllis J. Goodman, Patricia Hartge, Manal Hassan, Kathy Helzlsouer, Brian Henderson, Elizabeth A. Holly, Robert Hoover, Eric J. Jacobs, Aruna Kamineni, Alison Klein, Eric Klein, Laurence N. Kolonel, Donghui Li, Núria Malats, Satu Männistö, Marjorie L. McCullough, Sara H. Olson, Irene Orlow, Ulrike Peters, Gloria M. Petersen, Miquel Porta, Gianluca Severi, Xiao-Ou Shu, Kala Visvanathan, Emily White, Herbert Yu, Anne Zeleniuch-Jacquotte, Wei Zheng, Geoffrey S. Tobias, Dennis Maeder, Michelle Brotzman, Harvey Risch, Joshua N. Sampson, Rachael Z. Stolzenberg-Solomon

**Affiliations:** 1 Division of Cancer Epidemiology and Genetics, National Cancer Institute, Bethesda, Maryland, United States of America; 2 Information Management Systems, Inc., Calverton, Maryland, United States of America; 3 Yale School of Public Health/Yale Cancer Center, New Haven, Connecticut, United States of America; 4 Departments of Population Health, Obstetrics and Gynecology (Obs/Gyn) and Environmental Medicine, New York University, New York, New York, United States of America; 5 Department of Epidemiology, University of Washington, Seattle, Washington, United States of America; 6 Department of Epidemiology, Mayo Clinic, Rochester, Minnesota, United States of America; 7 Department of Epidemiology and Biostatistics, University of California San Francisco, San Francisco, California, United States of America; 8 Division of Cancer Epidemiology, German Cancer Research Center (DKFZ), Heidelberg, Germany; 9 Dalla Lana School of Public Health, University of Toronto; Prevention and Cancer Control, Cancer Care Ontario Toronto, Ontario, Canada; 10 Catalan Institute of Oncology (ICO-IDIBELL), Barcelona, Spain; 11 Samuel Lunenfeld Research Institute, Mount Sinai Hospital, University of Toronto, Toronto, Canada; 12 Cancer Epidemiology Centre, Cancer Council Victoria and Centre for MEGA Epidemiology, School of Population Health, the University of Melbourne, Melbourne, Australia; 13 Departments of Oncology, Pathology and Medicine, The Johns Hopkins University School of Medicine, Baltimore, Maryland, United States of America; 14 Cleveland Clinic, Glickman Urological and Kidney Institute, Cleveland, Ohio, United States of America; 15 Department of Gastrointestinal Medical Oncology, The University of Texas M. D. Anderson Cancer Center, Houston, Texas, United States of America; 16 MD Mercy, Baltimore, Maryland, United States of America; 17 Department of Preventative Medicine, School of Medicine, University of Southern California, Los Angeles, California, United States of America; 18 Epidemiology Research Program, American Cancer Society, Atlanta, Georgia, United States of America; 19 GroupHealth Research Institute, Seattle, Washington, United States of America; 20 University of Hawaii Cancer Center, Manoa, Hawaii, United States of America; 21 Molecular Pathology Program, Spanish National Cancer Research Center, Madrid, Spain; 22 National Institute for Health and Welfare, Department of Chronic Disease Prevention, Helsinki, Finland; 23 Department of Epidemiology and Biostatistics, Memorial Sloan-Kettering Cancer Center, New York, New York, United States of America; 24 Hospital del Mar Institute of Medical Research (IMIM), and School of Medicine, Barcelona Spain; 25 Division of Epidemiology, Department of Medicine, Vanderbilt Epidemiology Center, and Vanderbilt-Ingram Cancer Center, Vanderbilt University, Nashville, Tennessee, United States of America; 26 Cancer Genomics Research Laboratory, National Cancer Institute, Division of Cancer Epidemiology and Genetics, Leidos Biomedical Research, Inc., Frederick National Laboratory for Cancer Research, Frederick, Maryland, United States of America; 27 Westat, Rockville, Maryland, United States of America; National Health Research Institutes, TAIWAN

## Abstract

Evidence on the association between vitamin D status and pancreatic cancer risk is inconsistent. This inconsistency may be partially attributable to variation in vitamin D regulating genes. We selected 11 vitamin D-related genes (*GC*, *DHCR7*, *CYP2R1*, *VDR*, *CYP27B1*, *CYP24A1*, *CYP27A1*, *RXRA*, *CRP2*, *CASR* and *CUBN*) totaling 213 single nucleotide polymorphisms (SNPs), and examined associations with pancreatic adenocarcinoma. Our study included 3,583 pancreatic cancer cases and 7,053 controls from the genome-wide association studies of pancreatic cancer PanScans-I-III. We used the Adaptive Joint Test and the Adaptive Rank Truncated Product statistic for pathway and gene analyses, and unconditional logistic regression for SNP analyses, adjusting for age, sex, study and population stratification. We examined effect modification by circulating vitamin D concentration (≤50, >50 nmol/L) for the most significant SNPs using a subset of cohort cases (n = 713) and controls (n = 878). The vitamin D metabolic pathway was not associated with pancreatic cancer risk (p = 0.830). Of the individual genes, none were associated with pancreatic cancer risk at a significance level of p<0.05. SNPs near the *VDR* (rs2239186), *LRP2* (rs4668123), *CYP24A1* (rs2762932), *GC* (rs2282679), and *CUBN* (rs1810205) genes were the top SNPs associated with pancreatic cancer (p-values 0.008–0.037), but none were statistically significant after adjusting for multiple comparisons. Associations between these SNPs and pancreatic cancer were not modified by circulating concentrations of vitamin D. These findings do not support an association between vitamin D-related genes and pancreatic cancer risk. Future research should explore other pathways through which vitamin D status might be associated with pancreatic cancer risk.

## Introduction

Vitamin D signaling is of interest in relation to cancer because of its hypothesized role in inducing immune cell differentiation and inhibiting tumor proliferation and angiogenesis [1]. In humans, most vitamin D is synthesized endogenously via exposure of the skin to solar ultra-violet B radiation, which converts 7-dehyrocholesterol in skin to vitamin D. Small amounts come also from dietary sources such as fish or fortified dairy products and, in some populations, dietary supplements [2].

Some previous studies have suggested lower pancreatic cancer risk with proxy markers of higher vitamin D status. Ecologic studies, which are based on population averages rather than individual level data, have shown lower pancreatic cancer death rates in areas with more sun exposure in Spain [3], the United States [4,5], and Japan [6,7]. A large, prospective study that used a predicted estimate of vitamin D status based on five determinants of serum 25-hydroxyvitamin D (25(OH)D) (dietary and supplemental vitamin D, skin pigmentation, adiposity, geographic residence, and leisure activity) also found an inverse association with pancreatic cancer risk [8].

Serum 25(OH)D is the most widely used biomarker to assess vitamin D status in epidemiologic studies as it reflects both endogenous synthesis and dietary vitamin D intake [9]. However, previous studies evaluating measured circulating 25(OH)D concentrations with risk of pancreatic cancer show conflicting results. A large, pooled study of serum concentrations from eight cohorts as part of the Vitamin D Pooling Project (952 cases, 1,333 controls) reported increased pancreatic cancer risk with higher circulating vitamin D concentrations (odds ratio (OR) = 2.12, 95% confidence interval (CI)) comparing serum levels ≥100 nmol/L to the referent 50–75nmol/L [10]. In contrast, a nested case-control study pooling five prospective cohorts (451 cases, 1,167 controls) suggested an inverse association OR = 0.67, 95% CI 0.46–0.97 comparing plasma 25(OH)D quintiles (>81.05 to <45.64 nmol/L) and no association when using the categories employed in the Vitamin D Pooling Project [11].

Pathway-based analysis of GWAS can detect associations that might be missed by focusing on single loci or even genes [12]. To our knowledge only one previous population based case-control study (628 cases, 1,193 controls) evaluated associations between genetic variants related to vitamin D and pancreatic cancer, and reported no single-nucleotide polymorphism (SNP) associations after adjustment for multiple comparisons [13]. In the present study, we used data from 20 studies in PanScans I-III to examine 11 genes in the vitamin D metabolic pathway and 213 corresponding SNPs with risk of pancreatic cancer. In a subset of the cohorts we assessed effect measure modification by circulating vitamin D concentrations. Some of the samples used in this analysis overlap with those utilized in the Vitamin D Pooling Project [10]. We hypothesized that the contradictory evidence on circulating vitamin D and risk of pancreatic cancer might be explained by genetic variations in vitamin D-related genes and multiplicative interaction between circulating vitamin D and genetic variation.

## Materials and Methods

### Study Participants

We obtained data from the 20 studies in the PanScan collaboration who agreed to participate in this pathway analysis. PanScan phases I-III have been previously described [14–16]. Our primary analysis included genotype data from 10 cohort studies and 10 case control studies in the PanScan collaboration. Participating cohorts included the Agricultural Health Study, Alpha-Tocopherol, Beta-Carotene Cancer Prevention Study (ATBC), Give us a Clue to Cancer and Heart Disease Study (CLUE II), Cancer Prevention Study II (CPS-II), Melbourne Collaborative Cohort Study (MCCS), Multiethnic Cohort (MEC), New York University Women’s Health Study (NYU-WHS), Prostate Lung Colorectal and Ovarian Cancer Screening Trial (PLCO), Selenium and Vitamin E Cancer Prevention Trial (SELECT) and the VITamins and Lifestyle cohort (VITAL). The included case-control studies were the Mayo Clinic Molecular Epidemiology of Pancreatic Cancer Study, University of California San Francisco, Yale University, MD Anderson Cancer Center, University of Toronto, Johns Hopkins University, Memorial Sloan-Kettering Cancer Center, PACIFIC Study of Group Health and Northern California Kaiser Permanente, Spanish Pancreatic Cancer Study (PANKRAS II) [17], and PANcreatic Disease ReseArch (PANDoRA) (Heidelberg, Germany) [18]. All cases were diagnosed with primary pancreatic adenocarcinoma (ICD-O-3 code C250-C259 or **C25.0-C25.3, C25.7-C25.9**). In short, PanScans-I and II used a nested case-control study design for the cohort studies. Cohort cases were confirmed through cancer registries, death certificates or review of medical records by medical personnel. Cohort controls for PanScan-I were incidence density sampled with a 1:1 ratio and were alive and cancer free at the time of diagnosis of the matched case. In all case-control studies, matching criteria included calendar year of birth within five years, gender, race and ethnicity, while some cohorts also matched on age at baseline or blood draw, smoking, date/time of blood draw, fasting status at time of blood draw, and length of follow-up. All data was de-identified before genotyping and before samples were sent to NCI. Genotyping was performed at the National Cancer Institute’s (NCI’s) Cancer Genomics Research Laboratory (formerly known as the Core Genotyping Facility) using the Illumina HumanHap550 array for PanScan-I, the Illumina Human 610-Quad array for PanScan II, and the Illumina Human 770-Quad chip for PanScan III. In PanScan III controls were previously genotyped using second-generation Illumina SNP microarrays (e.g. OmniExpress, Omni 1M or Omni 2.5M) and drawn from PanScan III prospective cohorts and a Spanish case-control study and thus were not matched to the cases [19]. SNPs reported here were limited to those with minor allele frequencies (MAF) ≥5%. In total, we used data from 3,583 pancreatic cancer cases and 7,053 controls (1,108 cases and 4,353 controls from the cohorts and 2,475 cases and 2,700 controls from case-control studies) of European descent. The age and sex distribution of cases and controls is described in S1 Table. Written consent was obtained from all study participants. Each participating study was reviewed and approved by their local IRB for appropriateness in PanScan participation (S2 Table) [15].

### Vitamin D measurement

A subset of the cohort participants with GWAS data also had 25(OH)D measured in serum as part of the Vitamin D Pooling Project (713 cases and 878 controls) [20]. These subjects were from the following cohorts: ATBC, CLUE-II, CPS-II, NYU-WHS, and PLCO. Methods for assaying 25(OH)D have been previously described [10]. In short, Heartland Assays, Inc. (Ames, Iowa) performed assays for 25(OH)D for samples from CLUE-II, CPS-II, NYU-WHS, PLCO, and a subset of ATBC samples using the DiaSorin LIAISON 25 OH Vitamin D TOTAL Assay (Diasorin, Inc., Stillwater, Minnesota). The remaining ATBC samples were assayed previously using a similar method in the laboratory of Dr. R. Vieth [21]. The methods and coefficients of variation percentages for the blinded quality control samples of the Heartland 25(OH)D measures have been previously reported (10). Using a nested components of variance analysis with logarithmically transformed quality control measures across batches, the overall intra- and interbatch coefficients of variation were 16.5% and 4.7% for the previously assayed concentrations in the ATBC and PLCO studies, respectively.

### Vitamin D-related functions of included genes

*DHCR7* (DHC-7 reductase) converts pro-vitamin D_3_ (7-dehydrocholesterol) in the skin to cholesterol. Alternatively, pre-vitamin D is formed from 7-dehydrocholestrol following dermal UVB exposure. Vitamin D is also derived from diet or supplements in the form of cholecalciferol (D_3_) or ergocalciferol (D_2_). The vitamin D binding protein (DBP, also known as GC), transports provitamin D to the liver, as well as other vitamin D compounds to target tissues. In the liver 25-hydroxylases *CYP2R1* and *CYP27A1* convert vitamins D_2_ and D_3_ from diet and sun exposure to 25(OH)D (calcidiol), the major circulating vitamin D metabolite. Calcidiol is then converted to the active form 1,25(OH)_2_D_3_ (calcitriol) by 1α-hydroxylase (*CYP27B1*) in the kidney and other organs. 1α-hydroxylase (*CYP27B1*) is localized on the inner mitochondrial membrane where it produces the active form of vitamin D which binds the vitamin D receptor (*VDR)* with substantially higher affinity than 25(OH)D. The vitamin D binding protein, GC, transports vitamin D metabolites to target organs, where calcitriol binds to *VDR* and forms a heterodimer with *RXRA* (retinoid x-receptor alpha). This heterodimer attaches to vitamin D response elements on various target genes, some of which are thought to have anti-carcinogenic properties [1]. Vitamin D is catabolized by 24-hydroxylase (*CYP24A1*) to inactive forms [22].

Three additional genes, cubulin (*CUBN*), megalin (*LRP2)* and Calcium Sensing Receptor (*CASR*) were added to our analysis to be consistent with the previous study on vitamin D-related genes and pancreatic cancer [13]. Both cubulin and megalin are plasma membrane receptors that in combination mediate an endocytic update of GC-bound vitamin D. The *CASR* membrane protein binds calcium in the extracelluar matrix and plays an important role in calcium homeostasis.

In total, genotype data from 213 tag SNPs with MAF > 0.05 in 11 genes involved in the synthesis (*DHCR7*, *CYP27A1*, *CYP2R1*), transport (*GC*, *CASR*), metabolism (*CYP27B1*, *LRP2*, *CUBN*), signal transduction (*VDR*, *RXRA*) or catabolism (*CYP24A1*) of endogenous vitamin D were used in our analysis [1]. These SNPs are located within a span of 20kb 5′ upstream and 10kb 3′ downstream of the gene coding region as defined by the National Center for Biotechnology Information’s human genome build 36.3. SNPs and associated genes are listed in S3 Table.

### Statistical analysis

We used unconditional logistic regression to test the association between individual SNPs and pancreatic cancer risk, adjusting for age (≤50, 51–60, 61–70, 71–80, ≥81 years), sex, study, and 5 eigenvectors capturing ethnic ancestry. We performed the pathway and gene analyses using the R package AdaJoint and the adaptive rank truncated product (ARTP) statistic [23]. In each analysis 1,000,000 permutations were conducted. This statistic accounts for gene or pathway size and linkage disequilibrium and summarizes joint association signals within a gene or pathway. Analyses were restricted to Caucasians. We also tested for heterogeneity between the three phases (PanScan I, PanScan II and PanScan III) using the R package for fixed effects meta-analysis. We adjusted for multiple comparisons in the p-heterogeneity analysis considering a p-value<0.0002 as significant. Analyses stratified by the three PanScan phases were performed to explore possible differences in associations by phase.

To test possible effect modification by vitamin D status, we performed stratified analyses in the subset of cohort studies with measured 25(OH)D; we created a dichotomous variable for circulating vitamin D (≤50 nmol/L or >50 nmol/L), as above this threshold is considered to be adequate for bone and overall health in national recommendations [24,25] and was close to the median for controls in our study population (control median 25(OH)D = 51.5 nmol/L). In this subset analysis we additionally adjusted for smoking (never, former, current), body mass index (BMI), and season of blood draw (fall, winter, spring, summer). To test for multiplicative interaction, we created an interaction term between circulating vitamin D as a dichotomous variable and individual SNPs. Using a Bonferroni correction for multiple comparisons, genes with a p-value<0.006 and SNPs with a p-value<0.0002 were considered statistically significant. We additionally evaluated whether SNPs associated with vitamin D levels in published GWAS (rs2282679, rs12785878, rs10741657, rs6013897) on circulating vitamin D [26,27], or representative tag SNPs, were associated with vitamin D in a subset of our study sample.

## Results

Genetic variation in the vitamin D metabolic pathway overall was not associated with risk of pancreatic cancer (pathway ARTP p-value = 0.830, Table 1). None of the 11 genes were associated with pancreatic cancer (Table 1). SNPs near the *VDR* (rs2239186), *GC* (rs2282679), *LRP2* (rs4668123), *CYP24A1* (rs2762932), and *CUBN* (rs1810205) genes were the top SNPs associated with pancreatic cancer (p-values 0.008–0.037) (Table 2), although they did not reach the threshold for statistical significance after adjusting for multiple comparisons.

**Table 1:** Pathway analysis for risk of pancreatic cancer and gene sets in the vitamin D pathway (3,583 cases and 7,053 controls)

	SNPS (n)	Gene/pathway p-value^a^	Most significant SNP
**Total Pathway**	213	0.830	rs2239186
**Gene**			
*VDR*	22	0.116	rs2239186
*GC*	7	0.186	rs2282679
*LRP2*	33	0.328	rs4668123
*CYP24A1*	24	0.401	rs2762932
*CYP27B1*	3	0.457	rs10877013
*CASR*	13	0.568	rs7632399
*CYP2R1*	8	0.699	rs1562902
*CYP27A1*	5	0.704	rs7566656
*DHCR7*	4	0.873	rs3750997
*RXRA*	17	0.760	rs3132294
*CUBN*	77	0.798	rs1810205

^a^P-values account for number of SNPs within genes or within the overall pathway, but not for the total number of genes; Models were adjusted for age (≤50, 51–60, 61–70, 71–80, ≥81 years), sex, study and population stratification by 5 eigenvectors for ethnic ancestry.

**Table 2:** Vitamin-D related single nucleotide polymorphisms (SNPs) with p-values <0.05 and risk of pancreatic cancer from PanScan I-III (3,583 cases and 7,053 controls).

Gene	SNP	Chromosome	Alleles	MAF (case/control)	Nominal p-value^a^	Allelic OR^b^ ^,^ ^c^	Nominal P-heterogeneity by study phase^d^
*VDR*	rs2239186	12q13.11	T,C	0.192/0.208	0.008	0.89 (0.82–0.97)	0.914
	rs7967152	12q13.11	C,A	0.460/0.475	0.040	0.93 (0.87–1.00)	0.500
	rs12721364	12q13.11	C,T	0.142/0.152	0.046	0.91 (0.82–1.00)	0.740
*GC*	rs2282679	4q12-q13	A,C	0.270/0.287	0.036	0.92 (0.86–0.99)	0.176
*LRP2*	rs4668123	2q24-q31	C,T	0.250/0.272	0.027	0.90 (0.82–0.99)	0.002
*CYP24A1*	rs2762932	20q13	T,C	0.152/0.158	0.034	0.90 (0.82–0.99)	0.939
*CUBN*	rs1810205	10p12.31	A,G	0.384/0.378	0.037	1.08 (1.00–1.15)	0.043
	rs2356215	10p12.31	C,T	0.111/0.101	0.041	1.12 (1.00–1.25)	0.754

^a^ After bonferroni correction (0.05/213) p-values < 0.0002 were considered significant

^b^ Odds ratios (ORs) were adjusted for age (≤50, 51–60, 61–70, 71–80, ≥81 years), sex, study, and population stratification by 5 eigenvectors for ethnic ancestry.

^c^ Odds ratios (ORs) are for the number of copies of the minor allele.

^d^ Phase refers to participation in PanScan I, II or III. For rs4668123 data was available only from PanScan phases II and III.

A test for heterogeneity between the three phases of PanScan indicated no evidence of heterogeneity after adjustment for multiple comparisons. Results stratified by PanScan phase are presented for the overall pathway and 11 genes in S4 Table and for SNPs with nominal p-values <0.05 in S5 Table. No associations were significant after adjustment for multiple comparisons. In analyses stratified by high vs. low circulating vitamin D concentration, no significant differences were observed and tests for interaction between vitamin D concentration and each of the top 20 SNPs were not significant (all p-values > 0.1; data not shown).

Of the four variants identified as associated with circulating vitamin D concentration in published GWAS [26,27], in our sample only a tag SNP in *GC* showed an association (p = 5.30 x10^–7^) with vitamin D status; tag SNPs in or near *DHCR7/NADSYN1* and *CYP2R1* did not show an association and rs6013897 in *CYP24A1* could not be studied as the Illumina HumanHap550 platform does not include a tag SNP for rs6013897.

## Discussion

This study is the largest to date to evaluate the joint effects of SNPs in the vitamin D metabolic pathway and risk of pancreatic cancer. Contrary to our hypothesis that we would observe an association between the vitamin D metabolic pathway and risk of pancreatic cancer, we found no evidence for an association, either for the pathway, genes, individual SNPs, nor for interactions with measured serum vitamin D concentrations.

The previous study on genetic variants in the vitamin D pathway and pancreatic cancer risk based in Canada showed significant p-values for SNPs in the *CASR*, *CYP24A1*, *CYP2R1*, *DHCR7*, and *LRP2* genes (p-values ranged from 0.011–0.050), but after adjustment for multiple comparisons none of the associations remained significant [13]. Although for different SNPs, our results showing associations with SNPs in the *CYP24A1* and *LRP2* regions may offer some support these findings. The strongest SNP association demonstrated in our study was in the *VDR* gene (p-values 0.008–0.046 for three significant SNPs), which was not observed in the Canadian study.

Research specific to polymorphisms in the vitamin D metabolic pathway and pancreatic cancer is limited [28]. However, more research exists on the hormonally active form of vitamin D, calcitriol. Preclinical trials suggest anti-proliferative effects of calcitriol in skin, lymph nodes, and mammary tissues [29] and pancreatic cancer cells [30], attributed to mechanisms related to angiogenesis inhibition, G0/G1 cell cycle arrest, differentiation, induction of apoptosis and modulating different signaling pathways in tumor cells [1].

Strengths of our study include the very large sample size with genetic data, and the subset with both genetic data and 25(OH)D measured before diagnosis. Including genes in the pathway that not only have been shown to predict circulating vitamin D but also those that are known to be involved in metabolism in a collective pathway provides a broader scope of the biological process as it might relate to pancreatic cancer risk. Blood concentrations of vitamin D can vary by season of blood draw or other characteristics such as physical activity or BMI; although we did not have information on dietary and supplemental vitamin D intake, we adjusted for available covariates in the analyses stratified by circulating vitamin D status. Still, genetic data are less subject to influence or confounding by exogenous exposures. Limitations of our study include the differences between genotyping platforms, as 17 SNPs in the analysis were not included in the PanScan I platform. Also, although extensive quality control procedures were instituted, circulating 25(OH)D was assayed at two different locations, introducing the potential for batch effects or other variations between labs and measurements.

## Conclusion

Our findings do not support an association between common genetic variants in the vitamin D metabolic pathway and risk of pancreatic cancer, despite the large sample size and ability to assess effect measure modification by circulating vitamin D concentration. Future research should explore other pathways through which vitamin D might be associated with risk of pancreatic cancer; for example through studies of gene-nutrient interactions involving variants in downstream signaling pathways involving vitamin D.

## Supporting Information

S1 Table Sex and age distributions for cases and controls included in the analysis, separated by phase. (DOC)

S2 Table Included studies and associated Institutional Review Boards. (DOC)

S3 Table Complete list of SNPs (n = 213) and associated genes (n = 11) included in analysis. (DOC)

S4 Table Pathway analysis for risk of pancreatic cancer and gene sets in the vitamin D pathway, separated by PanScan phase. (DOC)

S5 Table Vitamin-D related single nucleotide polymorphisms (SNPs) with p-values <0.05 and risk of pancreatic cancer, separated by PanScan phase. (DOC)
