# Correction: Vitamin D Metabolic Pathway Genes and Pancreatic Cancer Risk

**DOI:** 10.1371/journal.pone.0129983

**Published:** 2015-06-03

**Authors:** Hannah Arem, Kai Yu, Xiaoqin Xiong, Kristin Moy, Neal D. Freedman, Susan T. Mayne, Demetrius Albanes, Laufey T. Amundadottir, Alan A. Arslan, Melissa Austin, William R. Bamlet, Laura Beane-Freeman, Paige Bracci, Federico Canzian, Stephen J Chanock, Michelle Cotterchio, Eric J. Duell, Steve Gallinger, Graham G. Giles, Michael Goggins, Phyllis J. Goodman, Patricia Hartge, Manal Hassan, Kathy Helzlsouer, Brian Henderson, Elizabeth A. Holly, Robert Hoover, Eric J. Jacobs, Aruna Kamineni, Alison Klein, Eric Klein, Laurence N. Kolonel, Donghui Li, Núria Malats, Satu Männistö, Marjorie L. McCullough, Sara H. Olson, Irene Orlow, Ulrike Peters, Gloria M. Petersen, Miquel Porta, Gianluca Severi, Xiao-Ou Shu, Stephen Van Den Eeden, Kala Visvanathan, Emily White, Herbert Yu, Anne Zeleniuch-Jacquotte, Wei Zheng, Geoffrey S. Tobias, Dennis Maeder, Michelle Brotzman, Harvey Risch, Joshua N. Sampson, Rachael Z. Stolzenberg-Solomon

Three authors are not included in the author byline.

Laufey T. Amundadottir should be listed as the 8^th^ author and affiliated with the Laboratory of Translational Genomics, Division of Cancer Epidemiology and Genetics, National Cancer Institute, National Institutes of Health, Bethesda, MD, USA. The contributions of this author are as follows: Performed the experiments, contributed reagents/materials/analysis/tools.

Stephen J Chanock should be listed as the 15^th^ author and affiliated with the National Cancer Institute, Division of Cancer Epidemiology & Genetics, Rockville. MD. The contributions of this author are as follows: performed the experiments, funded GWAS.

Stephen Van Den Eeden should be listed as the 44^th^ author and affiliated with the Kaiser Permanente Division of Research. 2000 Broadway, Oakland, CA 94612. The contributions of this author are as follows: conceived and designed the experiments, wrote the manuscript.
