# Supplementary material for: Vitamin D Metabolic Pathway Genes and Pancreatic Cancer Risk
Source: PLoS One. 2015 Mar 23;10(3):e0117574. doi: 10.1371/journal.pone.0117574 (PMC4370655; doi:10.1371/journal.pone.0117574)
Supplement: S1 Table — (DOC) [file pone.0117574.s001.doc]

**S1 Table.** Sex and age distributions for cases and controls included in the analysis

|  | **Cases, n** | |  |  | **Controls, n** | |  | |  | |
| --- | --- | --- | --- | --- | --- | --- | --- | --- | --- | --- |
|  | **Phase I^a^** | **Phase II^b^** | **Phase III^c^** | **Total** | **Phase I^a^** | **Phase II^b^** | | **Phase III^c^** | | **Total** |
| **Sex** |  |  |  |  |  |  |  | |  | |
| **Female** | 325 | 820 | 327 | 1472 | 329 | 876 | 529 | | 1734 | |
| **Male** | 607 | 940 | 495 | 2042 | 615 | 963 | 3664 | | 5242 | |
| **Age, years** |  |  |  |  |  |  |  | |  | |
| **<51** | 31 | 156 | 31 | 218 | 28 | 180 | 90 | | 298 | |
| **51-60** | 98 | 440 | 92 | 630 | 93 | 422 | 569 | | 1084 | |
| **61-70** | 347 | 602 | 234 | 1183 | 352 | 599 | 2141 | | 3092 | |
| **71-80** | 386 | 437 | 341 | 1164 | 402 | 521 | 1330 | | 2253 | |
| **>80** | 70 | 125 | 124 | 319 | 69 | 117 | 63 | | 249 | |

^a^PanScan phase I included 932 cases and 944 controls.

^b^PanScan phase II included 1,760 cases and 1,893 controls.

^c^PanScan phase III included 822 cases and 4,193 controls.
