# Supplementary material for: Vitamin D Metabolic Pathway Genes and Pancreatic Cancer Risk
Source: PLoS One. 2015 Mar 23;10(3):e0117574. doi: 10.1371/journal.pone.0117574 (PMC4370655; doi:10.1371/journal.pone.0117574)
Supplement: S2 Table — (DOC) [file pone.0117574.s002.doc]

**S2 Table**. Included studies and associated Institutional Review Boards

| **Study Name** | **Institutional Review Board (IRB)** |
| --- | --- |
| Agricultural Health Study | NCI Special Studies IRB |
| Alpha-Tocopherol, Beta-Carotene Cancer Prevention Study (ATBC) | NCI Special Studies IRB |
| Give us a Clue to Cancer and Heart Disease Study (CLUE II) | Johns Hopkins School of Public Health IRB |
| Cancer Prevention Study II (CPS-II) | Emory University IRB |
| Melbourne Collaborative Cohort Study (MCCS) | Cancer Council Victoria, Human Research Ethics Committee |
| Multiethnic Cohort (MEC) | University of Hawaii, Office of Research Compliance, Human Studies Program |
| New York University Women’s Health Study (NYU-WHS) | NYU School of Medicine IRB |
| Prostate Lung Colorectal and Ovarian Cancer Screening Trial (PLCO) | NCI Special Studies IRB |
| Selenium and Vitamin E Cancer Prevention Trial (SELECT) | SWOG IRB |
| VITamins and Lifestyle cohort (VITAL) | Fred Hutchinson Cancer Research Center IRB |
| Mayo Clinic Molecular Epidemiology of Pancreatic Cancer Study | Mayo Clinic IRB |
| University of California San Francisco (UCSF) | UCSF Human Research Protection Program, Committee on Human Research |
| Yale University | Yale University, Human Investigation Committee |
| MD Anderson Cancer Center (MDA) | MDA IRB |
| University of Toronto | Mount Sinai Hospital, Research Ethics Board |
| Johns Hopkins University (JHU) | JHU, Office of Human Subjects Research IRB |
| Memorial Sloan-Kettering Cancer Center (MSKCC) | MSKCC IRB/Privacy Board |
| PACIFIC Study of Group Health and Northern California Kaiser Permanente | Kaiser Permanente Northern California IRB |
| Spanish Pancreatic Cancer Study (PANKRAS II) | Ethical Committee of Clinical Research IRB of the Institut Municipal d’Assistencia Sanitaria |
| PANcreatic Disease ReseArch (PANDoRA) | Medizinische Fakultat Heidelberg , Ethickkommission |
