# Supplementary material for: Vitamin D Metabolic Pathway Genes and Pancreatic Cancer Risk
Source: PLoS One. 2015 Mar 23;10(3):e0117574. doi: 10.1371/journal.pone.0117574 (PMC4370655; doi:10.1371/journal.pone.0117574)
Supplement: S3 Table — (DOC) [file pone.0117574.s003.doc]

**Supplemental Table 3**. Complete list of SNPs (n=213) and associated genes (n=11) included in analysis;

| **SNP** | **GENE** |
| --- | --- |
| rs1814740 | CASR |
| rs4678126 | CASR |
| rs7632399 | CASR |
| rs1463890 | CASR |
| rs1354162 | CASR |
| rs4678029 | CASR |
| rs10934578 | CASR |
| rs9740 | CASR |
| rs1801725 | CASR |
| rs1393198 | CASR |
| rs2202127 | CASR |
| rs10222633 | CASR |
| rs3804592 | CASR |
| rs12258009 | CUBN |
| rs7087360 | CUBN |
| rs1810205 | CUBN |
| rs11254275 | CUBN |
| rs7912716 | CUBN |
| rs17431426 | CUBN |
| rs1907360 | CUBN |
| rs2271463 | CUBN |
| rs12255119 | CUBN |
| rs2291521 | CUBN |
| rs780806 | CUBN |
| rs12243895 | CUBN |
| rs1914172 | CUBN |
| rs12254816 | CUBN |
| rs7897550 | CUBN |
| rs11254325 | CUBN |
| rs17139378 | CUBN |
| rs780825 | CUBN |
| rs7072049 | CUBN |
| rs4748341 | CUBN |
| rs7900486 | CUBN |
| rs1276711 | CUBN |
| rs2356825 | CUBN |
| rs11254284 | CUBN |
| rs780807 | CUBN |
| rs12244831 | CUBN |
| rs4082518 | CUBN |
| rs4525114 | CUBN |
| rs3012479 | CUBN |
| rs2942359 | CUBN |
| rs2271461 | CUBN |
| rs10795445 | CUBN |
| rs7071576 | CUBN |
| rs1801239 | CUBN |
| rs703075 | CUBN |
| rs703062 | CUBN |
| rs12414709 | CUBN |
| rs9633766 | CUBN |
| rs11254308 | CUBN |
| rs12146414 | CUBN |
| rs1687716 | CUBN |
| rs780816 | CUBN |
| rs10904833 | CUBN |
| rs703065 | CUBN |
| rs7922682 | CUBN |
| rs1996316 | CUBN |
| rs12246329 | CUBN |
| rs2603804 | CUBN |
| rs12359150 | CUBN |
| rs2796833 | CUBN |
| rs10752062 | CUBN |
| rs7922356 | CUBN |
| rs7916688 | CUBN |
| rs2356215 | CUBN |
| rs11814420 | CUBN |
| rs11254339 | CUBN |
| rs11594134 | CUBN |
| rs7913144 | CUBN |
| rs1801241 | CUBN |
| rs9665553 | CUBN |
| rs11254336 | CUBN |
| rs11254351 | CUBN |
| rs780827 | CUBN |
| rs7913601 | CUBN |
| rs1801231 | CUBN |
| rs11591673 | CUBN |
| rs2145939 | CUBN |
| rs2883972 | CUBN |
| rs10904865 | CUBN |
| rs2271469 | CUBN |
| rs10904847 | CUBN |
| rs1801222 | CUBN |
| rs12766939 | CUBN |
| rs12261966 | CUBN |
| rs7082270 | CUBN |
| rs932640 | CUBN |
| rs780838 | CUBN |
| rs2585423 | CYP24A1 |
| rs927650 | CYP24A1 |
| rs4809960 | CYP24A1 |
| rs8124792 | CYP24A1 |
| rs1555439 | CYP24A1 |
| rs4809958 | CYP24A1 |
| rs912505 | CYP24A1 |
| rs765058 | CYP24A1 |
| rs4809959 | CYP24A1 |
| rs2248359 | CYP24A1 |
| rs6097797 | CYP24A1 |
| rs6068816 | CYP24A1 |
| rs6097801 | CYP24A1 |
| rs2762926 | CYP24A1 |
| rs2762932 | CYP24A1 |
| rs2762934 | CYP24A1^a^ |
| rs2585428 | CYP24A1^a^ |
| rs2296239 | CYP24A1^a^ |
| rs6022999 | CYP24A1^a^ |
| rs2245153 | CYP24A1^a^ |
| rs3787555 | CYP24A1^a^ |
| rs2181874 | CYP24A1^a^ |
| rs2296241 | CYP24A1^a^ |
| rs3886163 | CYP24A1^a^ |
| rs4674345 | CYP27A1 |
| rs4674338 | CYP27A1 |
| rs7566656 | CYP27A1^a^ |
| rs933994 | CYP27A1^a^ |
| rs1996992 | CYP27A1^a^ |
| rs10877013 | CYP27B1 |
| rs703842 | CYP27B1 |
| rs1048691 | CYP27B1 |
| rs11023374 | CYP2R1 |
| rs11023371 | CYP2R1 |
| rs16930625 | CYP2R1 |
| rs1993116 | CYP2R1 |
| rs2060793 | CYP2R1 |
| rs7935792 | CYP2R1 |
| rs1562902 | CYP2R1 |
| rs12794714 | CYP2R1^a^ |
| rs1790334 | DHCR7 |
| rs1790349 | DHCR7 |
| rs3750997 | DHCR7 |
| rs7944926 | DHCR7 |
| rs2282679 | GC |
| rs7041 | GC |
| rs705117 | GC |
| rs12512631 | GC |
| rs1352844 | GC |
| rs1491709 | GC |
| rs222020 | GC |
| rs2302696 | LRP2 |
| rs2284675 | LRP2 |
| rs10210408 | LRP2 |
| rs830983 | LRP2 |
| rs830956 | LRP2 |
| rs2241190 | LRP2 |
| rs10490131 | LRP2 |
| rs4667591 | LRP2 |
| rs9789747 | LRP2 |
| rs10490130 | LRP2 |
| rs700550 | LRP2 |
| rs831022 | LRP2 |
| rs853988 | LRP2 |
| rs861239 | LRP2 |
| rs12988804 | LRP2 |
| rs2239602 | LRP2 |
| rs830992 | LRP2 |
| rs2268375 | LRP2 |
| rs2892803 | LRP2 |
| rs7568568 | LRP2 |
| rs2673164 | LRP2 |
| rs2302694 | LRP2 |
| rs2247506 | LRP2 |
| rs10199676 | LRP2 |
| rs7592152 | LRP2 |
| rs831007 | LRP2 |
| rs3845732 | LRP2 |
| rs2161039 | LRP2 |
| rs2389557 | LRP2 |
| rs830998 | LRP2 |
| rs831023 | LRP2 |
| rs831010 | LRP2 |
| rs4668123 | LRP2^a^ |
| rs11185659 | RXRA |
| rs11102986 | RXRA |
| rs11185644 | RXRA |
| rs1805352 | RXRA |
| rs10881574 | RXRA |
| rs12339163 | RXRA |
| rs3118526 | RXRA |
| rs10881582 | RXRA |
| rs1805343 | RXRA |
| rs3118536 | RXRA |
| rs3818740 | RXRA |
| rs4917354 | RXRA |
| rs11185647 | RXRA |
| rs3118571 | RXRA |
| rs3132294 | RXRA |
| rs4240705 | RXRA |
| rs877954 | RXRA^a^ |
| rs2238136 | VDR |
| rs2853564 | VDR |
| rs4760648 | VDR |
| rs2254210 | VDR |
| rs1540339 | VDR |
| rs2239179 | VDR |
| rs7967152 | VDR |
| rs4237855 | VDR |
| rs1544410 | VDR |
| rs886441 | VDR |
| rs2239182 | VDR |
| rs2107301 | VDR |
| rs12721364 | VDR |
| rs4334089 | VDR |
| rs7968585 | VDR |
| rs4516035 | VDR |
| rs11574143 | VDR |
| rs2239186 | VDR |
| rs4328262 | VDR |
| rs2189480 | VDR |
| rs3890733 | VDR |
| rs9729 | VDR^a^ |

**^a^** These SNPs were available only in PanScans phases II and III
