# Supplementary material for: Vitamin D Metabolic Pathway Genes and Pancreatic Cancer Risk
Source: PLoS One. 2015 Mar 23;10(3):e0117574. doi: 10.1371/journal.pone.0117574 (PMC4370655; doi:10.1371/journal.pone.0117574)
Supplement: S4 Table — (DOC) [file pone.0117574.s004.doc]

**S4 Table. Pathway analysis for risk of pancreatic cancer and gene sets in the vitamin D pathway, separated by PanScan phase**

|  | **Phase 1^a^** |  | **Phase 2^b^** |  | **Phase 3^c^** |  | **Meta-analysis** | |
| --- | --- | --- | --- | --- | --- | --- | --- | --- |
|  | **SNPs (n)** | **p-value** | **SNPs (n)** | **p-value** | **SNPs (n)** | **p-value** | **SNPs (n)** | **p-value** |
| **Overall pathway** | 196 | 0.823 | 213 | 0.773 | 213 | 0.036 | 196 | 0.613 |
| **Gene** |  |  |  |  |  |  |  |  |
| ***VDR*** | 21 | 0.970 | 22 | 0.573 | 22 | 0.562 | 21 | 0.325 |
| ***GC*** | 7 | 0.519 | 7 | 0.774 | 7 | 0.085 | 7 | 0.145 |
| ***LRP2*** | 32 | 0.643 | 33 | 0.519 | 33 | 0.009 | 32 | 0.625 |
| ***CYP24A1*** | 15 | 0.873 | 24 | 0.757 | 24 | 0.159 | 15 | 0.185 |
| ***CYP27B1*** | 3 | 0.922 | 3 | 0.869 | 3 | 0.036 | 3 | 0.643 |
| ***CASR*** | 13 | 0.313 | 13 | 0.229 | 13 | 0.826 | 13 | 0.750 |
| ***CYP2R1*** | 7 | 0.237 | 8 | 0.156 | 8 | 0.233 | 7 | 0.145 |
| ***CYP27A1*** | 2 | 0.774 | 5 | 0.783 | 5 | 0.466 | 2 | 0.613 |
| ***DHCR7*** | 4 | 0.109 | 4 | 0.330 | 4 | 0.356 | 4 | 0.594 |
| ***RXRA*** | 16 | 0.937 | 17 | 0.417 | 17 | 0.579 | 16 | 0.735 |
| ***CUBN*** | 76 | 0.935 | 77 | 0.135 | 77 | 0.085 | 76 | 0.650 |

^a^PanScan phase I included (932 cases, 944 controls)

^b^PanScan phase II included (1,760 cases, 1,893 controls)

^c^PanScan phase III included (822 cases, 4,193 controls)
