# Supplementary material for: Vitamin D Metabolic Pathway Genes and Pancreatic Cancer Risk
Source: PLoS One. 2015 Mar 23;10(3):e0117574. doi: 10.1371/journal.pone.0117574 (PMC4370655; doi:10.1371/journal.pone.0117574)
Supplement: S5 Table — (DOC) [file pone.0117574.s005.doc]

**S5 Table. Vitamin-D related single nucleotide polymorphisms (SNPs) with p-values <0.05 and risk of pancreatic cancer, separated by PanScan phase**

| **Phase 1^a^** |  |  | **Phase 2^b^** |  |  | **Phase 3^c^** |  |  |
| --- | --- | --- | --- | --- | --- | --- | --- | --- |
| **Gene** | **SNP** | **p-value** | **Gene** | **SNP** | **p-value** | **Gene** | **SNP** | **p-value** |
| ***CASR*** | rs1814740 | 0.036 | CUBN | rs12258009 | 0.003 | LRP2 | rs4668123 | <0.001 |
| ***DHCR7*** | rs1790334 | 0.042 | CASR | rs7632399 | 0.025 | LRP2 | rs2302696 | <0.001 |
| ***LRP2*** | rs830983 | 0.047 | CYP2R1 | rs1562902 | 0.029 | LRP2 | rs2284675 | <0.001 |
|  |  |  | LRP2 | rs2673164 | 0.032 | CUBN | rs1810205 | 0.002 |
|  |  |  | CYP2R1 | rs1993116 | 0.045 | LRP2 | rs10210408 | 0.003 |
|  |  |  |  |  |  | CUBN | rs11254275 | 0.003 |
|  |  |  |  |  |  | LRP2 | rs10490131 | 0.010 |
|  |  |  |  |  |  | CYP24A1 | rs2585423 | 0.012 |
|  |  |  |  |  |  | LRP2 | rs4667591 | 0.012 |
|  |  |  |  |  |  | LRP2 | rs2241190 | 0.013 |
|  |  |  |  |  |  | GC | rs2282679 | 0.015 |
|  |  |  |  |  |  | CYP27B1 | rs10877013 | 0.018 |
|  |  |  |  |  |  | CYP27B1 | rs703842 | 0.021 |
|  |  |  |  |  |  | LRP2 | rs830956 | 0.024 |
|  |  |  |  |  |  | CYP24A1 | rs2585428 | 0.035 |
|  |  |  |  |  |  | CYP2R1 | rs11023374 | 0.046 |
|  |  |  |  |  |  | LRP2 | rs9789747 | 0.050 |

^a^PanScan phase I included 932 cases and 944 controls.

^b^PanScan phase II included 1,760 cases and 1,893 controls.

^c^PanScan phase III included 822 cases and 4,193 controls.
